# Clinical decision rules in the pre-hospital triage of patients with chest pain

**DOI:** 10.1007/s12471-023-01769-0

**Published:** 2023-03-14

**Authors:** Goaris W. A. Aarts, Peter Damman

**Affiliations:** Department of Cardiology, Radboud University Medical Centre, Nijmegen, The Netherlands

Overcrowding in the emergency department (ED) is a growing phenomenon worldwide, which is associated with increased length of hospital stay, high healthcare costs and worse patient outcomes. With the rapidly aging population in the Netherlands and frequent ED shutdowns due to staff shortages, this problem will probably not decrease in the near future. Chest pain is responsible for about 10% of all ED visits. It is important to rapidly diagnose or exclude life-threatening causes of chest pain. When an acute coronary syndrome (ACS) is suspected, the diagnostic work-up is a combination of a 12-lead electrocardiogram (ECG), clinical evaluation and cardiac troponin measurement(s) [1]. If the ECG shows signs of an ST-segment elevation myocardial infarction, the diagnosis is relatively straightforward. However, in more than 30% of the patients with a non-ST-segment elevation ACS (NSTE-ACS), the ECG is normal [1]. Therefore, chest pain patients with a suspected NSTE-ACS are often transported to the ED for further evaluation. However, 80–90% of the chest pain patients at the ED do not have an ACS [2]. Pre-hospital identification of patients without an ACS could therefore prevent unnecessary referrals, reduce overburdening of ambulance services and reduce ED overcrowding.

Multiple clinical decision rules to improve pre-hospital triage of chest pain patients have been developed and evaluated, both in the primary care setting and in the ambulance care setting. When patients contact the general practitioner or the emergency number, triage is initially performed by telephone consultation using standardised Netherlands Triage Standard (NTS) protocols. However, in chest pain patients the NTS leads to a high number of unnecessary referrals to the ED, while the level of urgency is underestimated in 27% of the patients with an ACS or other life-threatening conditions [3]. In this issue of the *Netherlands Heart Journal*, Manten et al. evaluate the performance of two clinical decision rules for telephone triage of chest pain patients contacting out-of-hours primary care facilities, the Marburg Heart Score (MHS) and the INTERCHEST score [4]. Both clinical decision rules are designed for ruling out coronary artery disease among primary care chest pain patients, based on risk scores incorporating the patient’s age, medical history and symptoms. In this study, the diagnostic capability of the NTS was modest at best for discriminating between patients with and without a major event (all-cause mortality, ACS, pulmonary embolism and other urgent conditions requiring hospital admission) or ACS. With thresholds for optimal diagnostic accuracy, the negative predictive value (NPV) and sensitivity were 97.2% and 78.6% for the MHS and 98.6% and 88.8% for the INTERCHEST score. The authors demonstrate that diagnostic risk stratification scores for chest pain improve telephone triage for major events in out-of-hours primary care, by reducing the number of unnecessary referrals without compromising triage safety. The next step in this setting will be the prospective validation of the use of these score-based clinical decision rules. We note that while both clinical decision rules outperformed the NTS by improving rule-out efficiency, their NPVs and sensitivities were lower than the minimal NPV and sensitivity of 99%, which are generally accepted for ruling out an ACS at the ED when using high-sensitivity cardiac troponin measurements [1]. With regard to the pre-hospital triage of chest pain patients, extensive research is currently being performed in the primary care and ambulance setting. Two of these studies include the HEART (History, ECG, Age, Risk factors and Troponin) score, which was initially designed for risk stratification at the ED [5]. The main difference between the HEART score and the previously described clinical decision rules is that the HEART score also includes the ECG, risk factors and a troponin measurement. The same group has shown previously that the use of a modified HEART score is useful in the primary care setting, increasing safety at the cost of doubling the number of patients referred to the ED [6]. Importantly, troponin measurements were not included in this study. A point-of-care (POC) troponin measurement allows the HEART score to be assessed by ambulance paramedics before transporting the patient to the hospital [7]. The recently published randomised ARTICA trial has shown that pre-hospital rule-out of NSTE-ACS in low-risk patients by HEART score assessment (in combination) with a low POC troponin concentration resulted in a reduction in transports to the ED, a reduction in healthcare costs, shorter time to ambulance availability and an incidence of ACS after 30 days which was comparable to that in patients for whom an ACS was ruled out at the ED [8]. Ruling out an NSTE-ACS in low-risk patients (HEART score ≤ 3 in combination with a low POC troponin concentration) had an NPV of 99.5% and sensitivity of 99.0% for ACS at 30 days.

In conclusion, these studies demonstrate that implementation of pre-hospital clinical decision rules has the potential to improve efficiency in the triage of chest pain patients, while reducing ED overcrowding. Moreover, ruling out an ACS in the pre-hospital setting with the HEART score in low-risk patients leads to a reduction in healthcare costs without increasing the number of patients with an ACS after 30 days, as compared to transporting all patients to the ED.

Novel developments include high-sensitivity POC troponin assays, the use of artificial intelligence to assist and expand pre-hospital ECG interpretation, and the introduction of regional pre-hospital triage networks with cardiologist videoconsultation options. These promising initiatives have the potential to further improve the diagnostic accuracy of the algorithms used and the logistics of care for chest pain patients.
